# Radiographic measurement of the congruence angle according to Merchant: validity, reproducibility, and limits

**DOI:** 10.1186/s43019-023-00175-5

**Published:** 2023-01-10

**Authors:** M. Severyns, J. Mallet, B. Santoni, T. Barnavon, A. Germaneau, T. Vendeuvre, M. Drame

**Affiliations:** 1 Department of Orthopaedic Surgery, Clinique Porte Océane, 85340 Les Sables d‘Olonne, France; 2 Pprime Institut UP 3346, CNRS, University of Poitiers, 86000 Poitiers, France; 3 Department of Clinical Research and Innovation, University Hospital of Martinique, 97200 Fort-de-France, France; 4 Hôpital Pierre Zobda Quitman, 97261 Fort-de-France Cedex, France

**Keywords:** Congruence angle, Femoro-patellar joint, Wiberg classification

## Abstract

**Purpose:**

The objective of this study was to analyze the intra- and interobserver variability of this measurement according to a strict methodology and on a representative sample of the general population, as well as to identify the possible difficulties of measurement in case of patellar or trochlear dysplasia.

**Methods:**

This observational study involved radiographic analysis by three independent observers of a total of 50 patients who had a loaded patellofemoral X-ray taken with the knee flexed to 45°. An initial reading was taken to measure the angle of the trochlear sulcus, the Merchant angle, and to classify the knees according to a possible trochlear dysplasia and/or patellar dysplasia according to Wiberg. A second measurement was then performed to analyze intraobserver agreement. Interobserver agreement was measured on all radiographic measurements (*n* = 100).

**Results:**

The Merchant patellofemoral congruence angle showed good intraobserver concordance ranging from 0.925 (95% CI 0.868–0.957) to 0.942 (95% CI 0.898–0.967), as well as interobserver concordance ranging from 0.795 (95% CI 0.695–0.862) to 0.914 (95% CI 0.872–0.942). Poor results were found in terms of interobserver concordance on the measurement of the Merchant angle in case of stage 3 Wiberg patella ranging from 0.282 (95% CI −0.920 to 0.731) to 0.611 (95% CI 0.226–0.892).

**Conclusion:**

Congruence angle is one of most commonly used measurements for patellar tracking. However, the convexity of the patellar surface makes it difficult to identify the patellar apex on its intraarticular facet, making the measurement of the Merchant congruence angle unreliable and not very reproducible in cases of stage 3 Wiberg patella.

*Registration* N°IRB 2021/139

## Introduction

Assessment of patellofemoral congruence is performed in routine practice in cases of patellofemoral instability [1, 2], painful patellar syndrome [3], or patellofemoral osteoarthritis [4]. In these cases, the analysis of patellofemoral congruence makes it possible to justify a surgical treatment choice in case of chronic instability or chronic patellofemoral pain (tibial tuberosity osteotomy, external patellar release, axial correction and/or derotation osteotomy, medial patellofemoral ligament reconstruction) [5–8]. In the literature, the Merchant angle or congruence angle is currently the most widely used radiographic measure for assessing this patellofemoral congruence [9–11]. However in 2013, a meta-analysis demonstrated that there was insufficient evidence to determine the reliability, validity, sensitivity, and specificity of this congruence angle [12]. In fact, the various studies found were conducted either on a population with healthy knees, representative of only 20% of the general population, or on series of cases of patellofemoral instability, with great variability in the radiographic acquisition protocols [2]. Moreover, no intraobserver or intraobserver analysis was performed of this radiographic measurement according to the level of trochlear or patellar dysplasia.

The main objective of this study was to analyze the intra- and interobserver variability of this radiographic angle of patellofemoral congruence described by Merchant according to a strict methodology and on a sample representative of the general population. The second objective was to analyze the variations of this angular measurement according to a possible patellar or trochlear dysplasia.

## Materials and methods

### Study population

This observational study allowed the radiographic analysis of a total of 50 patients who performed a loaded patellofemoral X-ray with the knee flexed to 45° according to the service protocol. These 50 radiological images were selected in chronological order of acquisition as of 1 January 2021, if all inclusion criteria were met. The inclusion criteria were: patient with closed physis and having performed a 45° patellofemoral X-ray under load on the native joint, and a profile X-ray to confirm the degree of flexion and to look for possible trochlear dysplasia. Patients with prosthetic or osteosynthesis material (*n* = 12), fractured patients (*n* = 4), and those who refused to participate in the study or who were under legal protection (*n* = 2) were excluded.

### Measurement method

**Fig. 1:**
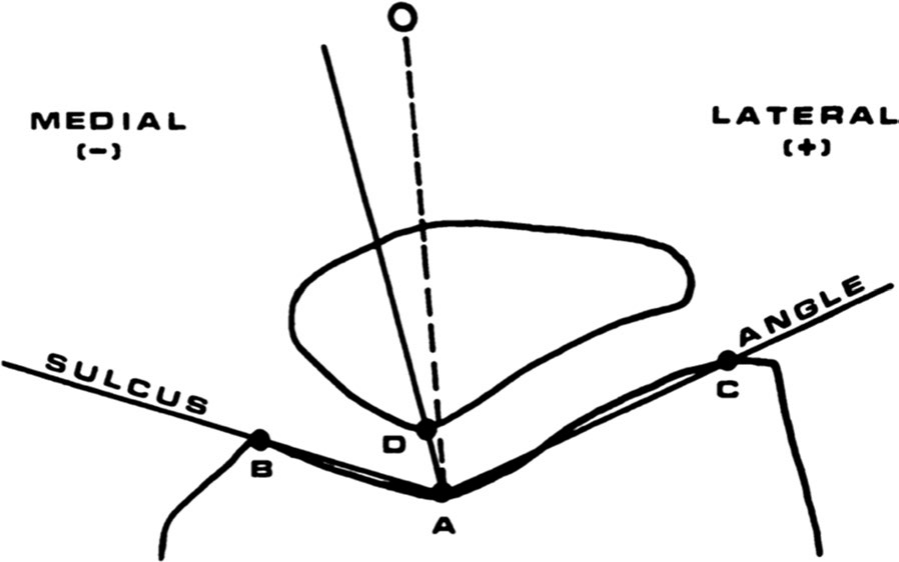
Method of measuring the patellofemoral congruence angle on a patellofemoral X-ray at 45° of flexion. Draw the angle of the sulcus (BAC) from the apex of the medial (**B**) and lateral (**C**) condyle, and identify the deepest point of the intercondylar sulcus (**A**). Draw the line AD through the apex of the sulcus angle (**A**) and the apex of the articular patellar ridge (**D**). Merchant angle is therefore the angle between the line DA and the bisector of the sulcus angle [9]

**Fig. 2:**
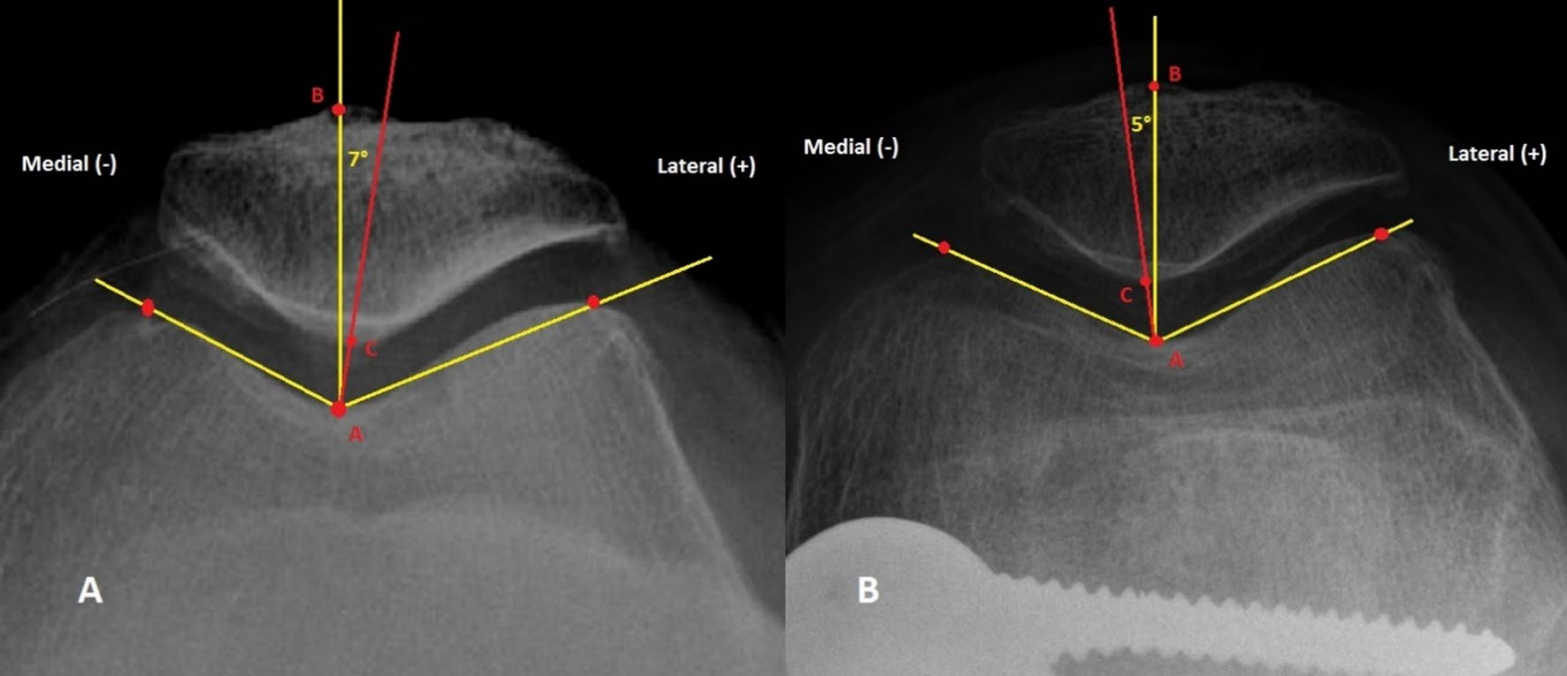
Example of measurement of the patellofemoral congruence angle according to Merchant on a patellofemoral image of the knee flexed at 45° ((**A**) native knee, (**B**) same knee after correction by distal femoral osteotomy)

**Fig. 3:**
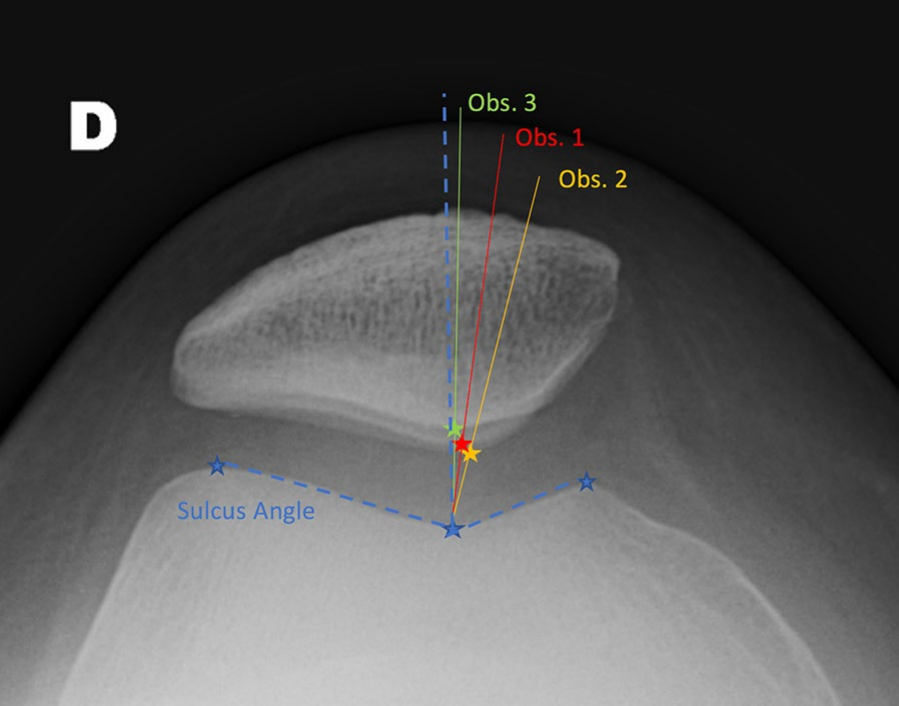
Radiograph of a Wiberg stage 3 patella case with intra- and interobserver variability in measurement

In 1974, Merchant et al. [9] proposed a method of assessing the degree of patellofemoral congruence by radiographic measurement of the patellofemoral congruence angle. To this day, this angle remains the reference radiographic method for measuring this congruence. To make this measurement (Fig. 1), the bisector of the sulcus angle is drawn to establish a zero reference line. A second line is then projected from the apex of the sulcus angle to the lowest point of the apex of the intraarticular facet of the patella. The angle measured between these two lines is the congruence angle described by Merchant [9]. If the apex of the patellar joint ridge is lateral to the zero line, then the congruence angle is positive. If it is medial, then the angle is negative (Figs. 2, 3).

### Assessment criteria

Radiographic analysis was performed independently by three senior orthopedic surgeons (J.M., T.B., B.S.) of which only one is specialized in the management of the patellofemoral joint (J.M.). All patellofemoral X-rays were analyzed. After anonymization of the radiographs, a first reading allowed each observer to measure the angle of the trochlear sulcus as well as the Merchant angle. A second measurement, still anonymized and in disorder, was performed to analyze the intraobserver concordance of the measurement of the Merchant congruence angle between all the participants taken in pairs. Interobserver concordance was measured on all radiographic measurements, i.e., a total of 100 radiographs. To analyze the intra- and interobserver variability according to a possible patellar or trochlear dysplasia that could complicate the measurements, an analysis of the intra- and interobserver concordances of the Merchant angle in subgroups was performed. The degree of trochlear dysplasia according to Dejour as well as the classification of the patella according to Wiberg were analyzed after the first measurements during a collegial meeting that allowed a consensus to be reached between the three observers on all the X-rays.

In case of poor agreement on the measurement of Merchant angle, a comparison was made with the measurement of the trochlear sulcus. This made it possible to identify the source of confusion on the radiographic measurement between the positioning of the marker on the apex of the patella or the measurement of the angle of the sulcus by the positioning of the markers on the tops of the condyles in coronal section.

### Statistical analysis

Data were collected in an Excel spreadsheet (Microsoft, Richmond, WA, USA) and analyzed with IBM SPSS software release 21 (IBM Corporation, Armonk, NY, USA) via a protocol validated by the institutional review board (IRB) dependent on the research department of our institution (IRB reference no. 2021/139). Continuous variables were described by their mean and standard deviation; and categorical variables by their number and percentage.

Intra- and interobserver agreement of Merchant angle measurement as well as trochlear sulcus angle was measured by the intraclass correlation coefficient, with its 95% confidence interval. In accordance with Nunnally and Berstein [13], an intraclass correlation coefficient greater than 0.7 could be considered good.

## Results

Table 1 presents the distribution of the different patella dysplasia according to the Wiberg classification as well as possible trochlear dysplasia among the 100 radiographic cases studied.

**Table 1:** Distribution of patients according to the Wiberg and Dejour classification (*N* = 50)

Classification	*n*	%
Wiberg (patellar dysplasia)		
1	8	8
2	74	74
3	18	18
Dejour (trochlear dysplasia)		
No	82	82
Yes*	18	18
A	2	2
B	4	4
C	8	8
D	4	4

*The 18 patients with trochlear dysplasia are then divided according to the type of dysplasia according to Dejour (A, B, C, or D)

Intra- and interobserver correlations of the Merchant angle, the degree of trochlear dysplasia, and patellar dysplasia are presented in Tables 2, 3, and 4.

**Table 2:** Intra-class correlation coefficients for Merchant congruence angle measures

	Intraclass correlation coefficient	CI 95%
Intraobserver concordance		
Obs. 1	0.943	0.892–0.965
Obs. 2	0.942	0.901–0.967
Obs. 3	0.926	0.869–0.956
Interobserver concordance		
Obs. 1/Obs. 2	0.797	0.690–0.865
Obs. 1/Obs. 3	0.859	0.791–0.911
Obs. 2/Obs. 3	0.912	0.870–0.945

**Table 3:** Results of the measurement of intra- and interobserver concordance according to the degree of trochlear dysplasia according to Dejour

	Trochlear dysplasia (*n* = 18)		No trochlear dysplasia (*n* = 82)	
	Intraclass correlation coefficient	CI 95%	Intraclass correlation coefficient	CI 95%
Intraobserver concordance				
Obs. 1	0.940	0.726–0.983	0.954	0.918–0.979
Obs. 2	0.825	0.216–0.954	0.962	0.922–0.966
Obs. 3	0.988	0.945–0.995	0.908	0.833–0.956
Interobserver concordance				
Obs. 1/Obs. 2	0.752	0.320–0.912	0.946	0.895–0.964
Obs. 1/Obs. 3	0.736	0.410–0.897	0.962	0.923–0.972
Obs. 2/Obs. 3	0.939	0.830–0.975	0.916	0.837–0.950

**Table 4:** Results of the measurement of intra- and interobserver concordance based on the Wiberg classification of the patella

	Wiberg 1 (*n* = 8)		Wiberg 2 (*n* = 74)		Wiberg 3 (*n* = 18)	
	ICC	CI 95%	ICC	CI 95%	ICC	CI 95%
Intraobserver concordance						
Obs. 1	0.812	− 1.961–0.990	0.954	0.912–0.979	0.686	0.380–0.937
Obs. 2	0.972	0.512–0.997	0.952	0.894–0.971	0.917	0.635–0.987
Obs. 3	0.890	− 0.684–0.932	0.937	0.890–0.972	0.759	0.020–0.953
Interobserver concordance						
Obs. 1/Obs. 2	0.806	0.028–0.961	0.848	0.742–0.914	0.285	(−0.918 to 0.738)
Obs. 1/Obs. 3	0.752	0.159–0.941	0.891	0.817–0.943	0.532	(−0.268 to 0.819)
Obs. 2/Obs. 3	0.905	0.517–0.975	0.942	0.902–0.958	0.608	(0.222–0.897)

In all the patients included, the intraobserver concordance of the Merchant angle was very good, ranging from 0.926 (95% CI 0.869–0.956) to 0.943 (95% CI 0.892–0.965). They were also good for interobserver concordance, ranging from 0.797 (CI 95% 0.690–0.865) to 0.912 (CI 95% 0.870–0.945).

All these results remained good with and without trochlear dysplasia (Table 3).

Regarding the Wiberg classification, poor results were found in terms of interobserver concordance on the measurement of the Merchant angle in case of Wiberg patella stage 3 ranging from 0.285 (95% CI −0.918 to 0.738) to 0.608 (95% CI 0.222–0.897).

Table 5 presents the results of the intraclass correlation of the angular measurements of the sulcus in case of stage 3 Wiberg patella. Since the results of this angular measurement are very satisfactory (ICCC of 0.922–0.941), the positioning of the landmark on the apex of the patella seems to be the cause of the poor results observed on the intraclass correlation measurements in case of stage 3 Wiberg patella.

**Table 5:** Evaluation of trochlea sulcus measurement by measuring the intraclass correlation coefficient

Wiberg 3 patella	Sulcus measurement (°)	
	Intraclass correlation coefficient	CI 95%
Interobserver concordance		
Obs. 1/Obs. 2	0.922	0.787–0.963
Obs. 1/Obs. 3	0.928	0.802–0.959
Obs. 2/Obs. 3	0.941	0.842–0.958

## Discussion

This study showed very good results in terms of intra- and interobserver concordance regarding the radiographic measurement of patellofemoral congruence angle. This corresponds to the results observed in the literature on the validity and reproducibility of this measurement in a population of patients with patellar instability, with better results by radiographic evaluation method than by computed tomography (CT) scan or MRI measurement [12].

Although it is a useful two-dimensional radiographic measurement to characterize the geometry of the patellofemoral joint, some authors feel that it provides limited information regarding the complex interface of the contact surfaces or the transmission of load through the patellofemoral joint compared with CT or MRI measurements. Recently, Lee et al. [14] established threshold values for patellofemoral parameters after three-dimensional CT reconstruction, including the congruence angle. Although these values facilitate the diagnosis and treatment planning of patellofemoral disorders in skeletally mature patients, their acquisition modalities remain complex, and were performed in the supine position with 15° of knee flexion. Regarding the evaluation and measurement by MRI, Ye et al. [15] found very poor interobserver reliability regarding the measurement of this congruence angle (ICCs 0.325–0.380), in contrast to the other measurements of the patellofemoral joint: Fulkerson angle [16], Laurin angle [17], patellar tilt angle (PTA), lateral patellar displacement (LPD), and bisect offset ratio (ICCs > 0.8). For the authors, these poor results could be explained by the changes in knee position during iconographic acquisition and/or the difficulty in identifying the patellar apex [15].

This last finding can be correlated with the results of our secondary objective. Poor results in terms of intra- and interobserver concordance were found in cases of stage 3 Wiberg patella, whereas the sulcus measurement obtained an intraclass correlation coefficient greater than 0.7. The convexity of the patella facet joint makes radiographic measurement of the Merchant congruence angle difficult and not reproducible. However, patients with Wiberg stage 3 patellar joints appear to be at greater risk of patellofemoral osteoarthritis and therefore require a radiographic evaluation [18].

While the reference patellofemoral X-ray protocol at 45° of flexion described by Merchant should be performed in the supine position, imaging protocols tend to perform these same acquisitions in the weight-bearing position with the same reliability [19]. In this work, the authors wanted to be in loaded conditions. Indeed, the movement of the patella in its patellofemoral joint is affected by the contraction of the quadriceps muscle [20] as well as the joint load [21, 22]. Compared with the supine position protocol, the 45° loaded patellofemoral X-ray would allow for a decrease in the congruence angle [23]. Consideration of both loaded and unloaded images appears to provide additional information in the radiographic evaluation of the patellofemoral joint.

This study has a number of limitations related primarily to its retrospective nature. Our study did not allow us to ensure the correct positioning of the knee at 45° during the various acquisitions. In their systematic review, Nord et al. [24] observed great heterogeneity in imaging protocols and poor reproducibility of axial patellofemoral X-rays. Although this was not detrimental to our results, this variability could lead to a disparity in the results of radiographic images used for clinical decision making [24].

In our study, all three observers were senior orthopedic surgeons but only one was experienced in patellofemoral radiological analysis. For a large number of authors [25, 26], the level of experience seems to play an important role in the evaluation and measurement of patellofemoral parameters with a significant learning curve. As an example, Smith et al. [25] found intraobserver variations in congruence angle measurement that could range from 0.8° to 18.4° for the same imaging. These variations tended to balance out according to the observer’s level of experience. Although the results were similar between the “naïve” and the “expert” observers, This notion should be taken into consideration when analyzing the results of this work which were initially measured by three senior orthopedic surgeons.

## Conclusion

Congruence angle is one of most commonly used measurements for patellar tracking. The patellofemoral congruence angle according to Merchant shows good intra- and interobserver concordance in a population with and without patellofemoral dysplasia, apart from cases of Wiberg stage 3 patella. Indeed, the convexity of the patellar surface makes it difficult to identify the patellar apex on its intraarticular facet, making the measurement of this angle unreliable and not very reproducible in this case.

## Data Availability

Data will not be shared.
